# Detailed examination of a double-dividend case in a recent two-engine growth model

**DOI:** 10.1016/j.mex.2025.103562

**Published:** 2025-08-11

**Authors:** Kei Hosoya

**Affiliations:** Faculty of Economics, Kokugakuin University, 4–10–28 Higashi, Shibuya-ku, Tokyo 150–8440, Japan

**Keywords:** Multiple growth engines, Economic growth, Environmental quality improvements

## Abstract

An important recent achievement in environmental macroeconomics is that reported by Cheng et al. [J. Macroecon. 81, 103614 (2024)]. Their model implements multiple growth engines and realizes the double dividend of economic growth and environmental improvements. However, regarding the environmental aspect, the situation could change drastically in general cases, and so a detailed understanding of the mechanisms in such cases is important for future similar studies. This paper presents additional numerical results that complement the analysis by Cheng et al. (2024), clarifies the underlying mechanisms, and makes some policy comments.

## Review highlights

· This paper examines in detail some of the results in the paper by Cheng et al. (2024), which is likely to become an important basic reference in environmental macroeconomics.
· While Cheng et al. (2024) clarified the realization of a double dividend with respect to growth and the environment, the mechanism of their model is complex, with environmental degradation seen in some cases as judged by pollution emissions.
· In this context, we use numerical analyses to explore their model in depth.

## Graphical abstract

**Figure:**
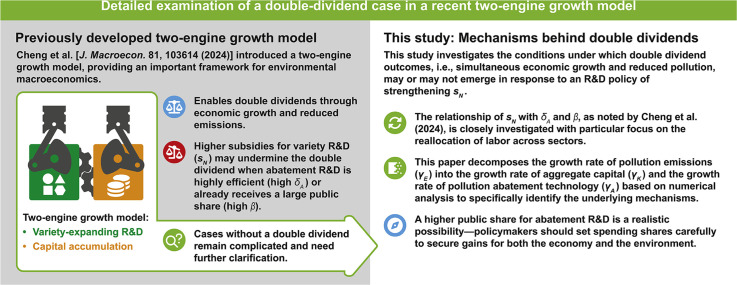

**Specifications table**

**Table:** 

**Subject area**	Economics and Finance
**More specific subject area**	Macroeconomic dynamics with environmental factors
**Name of the reviewed methodology**	R&D-based growth model with numerical computations
**Keywords**	Multiple growth engines; Economic growth; Environmental quality improvements
**Resource availability**	None
**Review question**	Regarding the main argument by Cheng et al. (2024) for realizing a double dividend, this paper focuses particularly on cases where no such dividend can be obtained because of environmental degradation and clarifies what happens in the model through graphical illustrations based on numerical analysis.

## Background

In an interesting study, Cheng et al. [1] focused on the two growth engines of variety-expanding research and development (R&D) in line with Romer [2] and Jones [3] and capital accumulation as driving forces of economic growth.^1^ A further feature of this model is that it also accounts for environmental factors, including abatement R&D. In the model, a patent strengthening policy and multiple subsidy programs are considered as growth-promoting measures, and their specific impacts, which of course also affect the environment, are identified through comprehensive numerical analysis. A good situation is when a country enjoys the so-called “double dividend” of promoting economic growth and improving the environment, but even in such a case, pollution emissions can be encouraged in the opposite direction when either the environmental abatement R&D sector (abatement labor productivity) is highly efficient or the share of expenditure on abatement R&D is high. Cheng et al. [1] provided only a somewhat facile analysis and brief discussion of the causes of this situation, and it is not necessarily easy to glimpse what is occurring inside their model. Because the paper by Cheng et al. [1] is likely to be cited as an important reference in the future, we seek to clarify this important issue in depth. As such, this paper focuses on two cases in which pollution emissions increase when the subsidy rate to variety-expanding R&D is increased, and as a result the aforementioned double dividend is no longer realized. We then present additional numerical results and discuss these cases in detail.

Incidentally, as in Cheng et al. [1], the R&D-based growth framework has become increasingly common in the recent environmental macroeconomics literature (e.g., [[4], [5], [6], [7], [8], [9], [10], [11]]). Among these, Chu and Lai [12] is significant achievement, particularly in terms of the sophistication of its dynamic macroeconomic model and its rich implications for environmental policy.^2^ They designed three different schemes for abatement R&D activities and subsidizing R&D with environmental tax revenue, and compared their impact on economic and environmental performance. The results showed that when intermediate goods firms have sufficient monopoly power, a mixed abatement policy of publicly subsidizing private R&D activities with environmental tax revenue is most favorable for the public welfare, as affected by economic growth and environmental quality.

The idea of R&D-based growth not only explains the creation of new products as an engine of growth but also enables the description of environmental R&D activities aimed at reducing environmental impact. Therefore, this type of analytical framework is generally useful for two-engine growth models.

## Method details

### Supplementary notes to the numerical results on pollution emissions in Cheng et al. [1]

The variables and parameters appearing in this paper are summarized in Table 1.

**Table 1:** List of variables and parameters.

$\gamma_{Y}\equiv \dot{Y}/Y$	growth rate of final output
$\gamma_{E}\equiv \overset{˙}{E}/E$	growth rate of pollution
$s_{N}$	subsidy rate on variety R&D
L	total labor force
Δ	$\equiv \rho /\delta_{N}+\rho /\delta_{K}$
$\delta_{K}$	productivity of the capital-producing sector
α	input share of intermediate goods in the final goods production
$\delta_{A}$	productivity of the abatement R&D sector
β	share of public expenditure on (publicly) abatement R&D as a percentage of GDP
μ	degree of patent breadth
$s_{K}$	subsidy rate on capital goods production
Ψ	$\equiv \alpha \left[\mu \left(1-s_{K}\right)+s_{K}-s_{N}\right]+\mu \left(1-s_{N}\right)\left(1-s_{K}\right)\left(1-\alpha +\beta \right)$
ρ	subjective discount rate
$L_{A}$	labor input employed in the abatement R&D sector
$\delta_{N}$	productivity of the variety R&D sector
$\gamma_{K}\equiv \overset{˙}{K}/K$	growth rate of capital
$\gamma_{A}\equiv \overset{˙}{A}/A$	growth rate of pollution abatement technology (knowledge)
η	extent of households’ environmental consciousness in instantaneous utility function

Of particular importance to Cheng et al. [1] for enjoying a double dividend were the dynamics involved in environmental quality.^3^ Specifically, if the growth of polluting emissions, which are by-products of intermediate goods production, is controlled, then the overall environmental quality improves (on the other hand, the increase in intermediate goods contributes to economic growth). First, the dynamic equation for pollution emissions ($\overset{˙}{E}/E$) is

(1) $$\frac{\overset{˙}{E}}{E}\equiv \gamma_{E}=\frac{\left(1-s_{N}\right)\left(L+\Delta \right)\left[\delta_{K}\alpha -\delta_{A}\beta \mu \left(1-s_{K}\right)\right]}{\Psi }-\rho ,\;$$

where we define $\Psi \equiv \alpha \left[\mu \left(1-s_{K}\right)+s_{K}-s_{N}\right]+\mu \left(1-s_{N}\right)\left(1-s_{K}\right)\left(1-\alpha +\beta \right)$.^4^ Cheng et al. [1] focused on the characteristic case in which the government provides growth-promoting policies at different subsidy rates ($s_{N}\neq s_{K}$) to the variety-expanding R&D sector devoted to expanding the variety of intermediate goods and to the capital goods production sector.^5^ Applying different subsidy rates is natural given the different characteristics of each sector. Among such cases, that with a positive subsidy rate for variety-expanding R&D and no subsidy for capital goods production ($s_{N}>0,\;s_{K}=0$) is particularly worth considering and is also treated in detail herein.

From (1), we obtain

(2) $$\frac{\partial \gamma_{E}}{\partial s_{N}}=\frac{\alpha \left(\mu -1\right)\left(1-s_{K}\right)\left(L+\Delta \right)\left[\beta \mu \left(1-s_{K}\right)\delta_{A}-\alpha \delta_{K}\right]}{\Psi^{2}},$$

the sign of which is never simply determined. The square-bracket part of the numerator is indeterminate, and thus the result is classified as follows:

(3) ∂γE∂sN><0⇔ifδA><αδKβμ(1−sK).

As is clear from (3), the theoretical condition for a decrease in pollution emissions is that the production efficiency of the abatement R&D sector is not too high and must be less than $\frac{\alpha \delta_{K}}{\beta \mu (1-s_{K})}$.

Increasing the R&D subsidy rate $s_{N}$ induces sectoral mobility of workers/researchers and their concentration from other sectors to the variety-expanding R&D sector. According to Cheng et al. [1], two different effects arise in this case: the benefit effect is that labor mobility slows the production of capital goods (inputs for the production of intermediate goods) related to pollution emissions, thus reducing pollution. In contrast, the cost effect is that as abatement labor is decreased, so the development of abatement technology slows, thereby promoting pollution. In the benchmark case, the former outweighs the latter, and the pollution growth rate $\gamma_{E}$ is decreasing with respect to the subsidy rate to variety-expanding R&D ($s_{N}$). However, when the efficiency of the abatement R&D sector ($\delta_{A}$) and the expenditure ratio on public abatement R&D activity (β) are relatively high, the observations in the benchmark case are overturned. We now examine these details below.

First, we consider the case with high $\delta_{A}$. Cheng et al. [1] presented three cases, namely, $\delta_{A}=\left\{0.95,\;1.95,\;2.95\right\}$, and at $\delta_{A}=2.95$, $\gamma_{E}$ turns into an increasing function of $s_{N}$. That is, the *story* is that when the productivity of abatement labor (the production efficiency of abatement R&D) is relatively high, an increase in subsidy to variety-expanding R&D simultaneously causes more pollution by substantially reducing abatement labor.

To verify the details, we observe the behavior of abatement labor $L_{A}$, which is given by

(4) $$L_{A}=\frac{\beta \mu \left(1-s_{N}\right)\left(1-s_{K}\right)\left(L+\Delta \right)}{\Psi }.$$

From (4), note that β but not $\delta_{A}$ directly affects the relationship between $L_{A}$ and $s_{N}$ (neither Ψ nor Δ includes $\delta_{A}$, of course).^6^

**Fig. 1:**
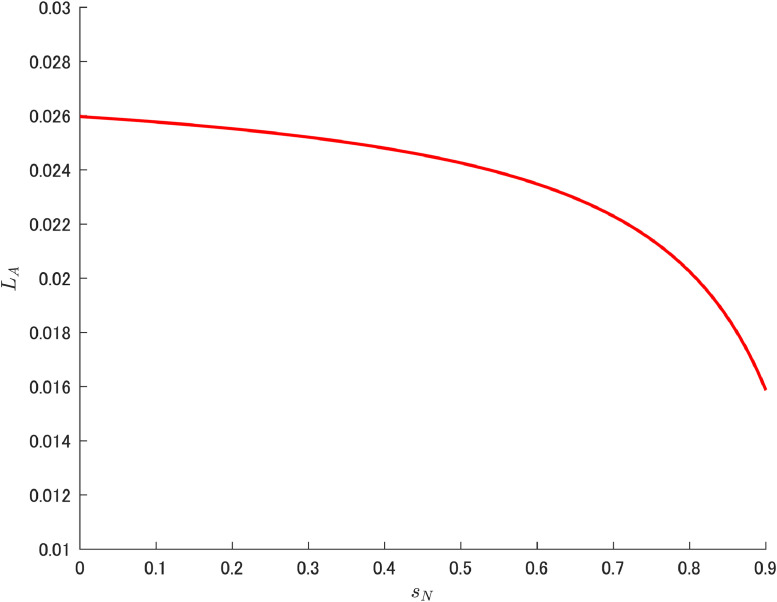
Relationship between R&D subsidy and abatement labor. Note: Author’s calculation.

Table 2 gives each parameter used by Cheng et al. [1], under which $L_{A}$ can be drawn as in Fig. 1. This downward shape is consistent with the result confirmed theoretically by Cheng et al. [1].^7^ Specifically, the following was obtained by assuming μ>1, which is a parameter representing the degree of patent breadth as determined by the government:

(5) $$\frac{\partial L_{A}}{\partial s_{N}}=\frac{-\alpha \beta \mu \left(L+\Delta \right){\left(1-s_{K}\right)}^{2}\left(\mu -1\right)}{\Psi^{2}}<0.$$

Again, note that the amount of abatement labor is not directly related to $\delta_{A}$.

**Table 2:** Baseline parameters.

Parameter	Value	Parameter	Value
ρ	0.04	$\delta_{N}$	0.4699
$\delta_{K}$	0.1671	$\delta_{A}$	1.95
α	0.4	$s_{K}$	0
L	1	μ	1.22
β	0.02		

Note: Based on Cheng et al. [1].

In this context, what somewhat complicates the understanding of the resulting situation is the effect of labor reallocation, especially the fact that $\delta_{A}$ itself has no effect on abatement R&D labor. On the other hand, the resulting pollution growth rate $\gamma_{E}$ is affected by $\delta_{A}$. With respect to how $\delta_{A}$ acts to transform the pattern of $\gamma_{E}$, it is informative to check the composition of $\gamma_{E}$. Given that the relation $\gamma_{E}=\gamma_{K}-\gamma_{A}$, $\gamma_{K}$, and $\gamma_{A}$ can be expressed as

$$\gamma_{K}=\frac{\delta_{K}\alpha \left(1-s_{N}\right)\left(L+\Delta \right)}{\Psi }-\rho ,$$

$$\gamma_{A}=\frac{\delta_{A}\beta \mu \left(1-s_{N}\right)\left(1-s_{K}\right)\left(L+\Delta \right)}{\Psi }.$$

**Fig. 2:**
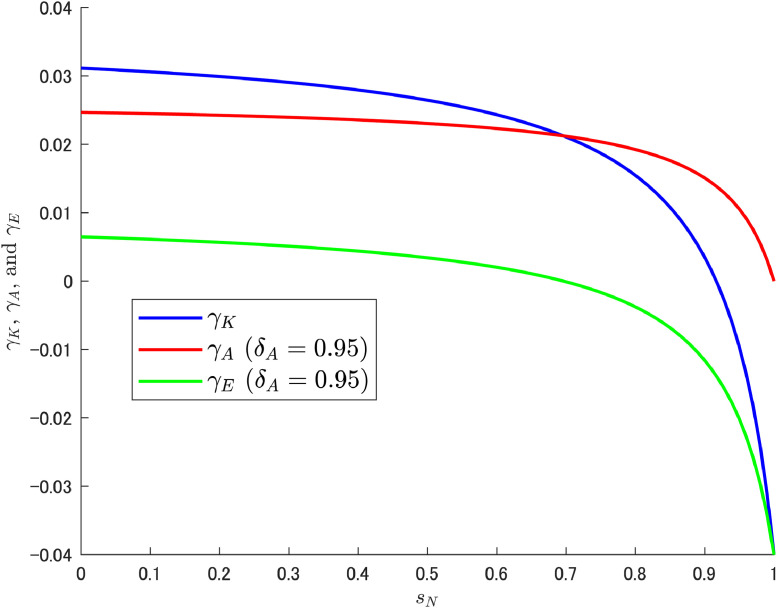
Relationship among $\gamma_{K}$, $\gamma_{A}$, and $\gamma_{E}$ under $\delta_{A}=0.95$. Note: Author’s calculation.

**Fig. 3:**
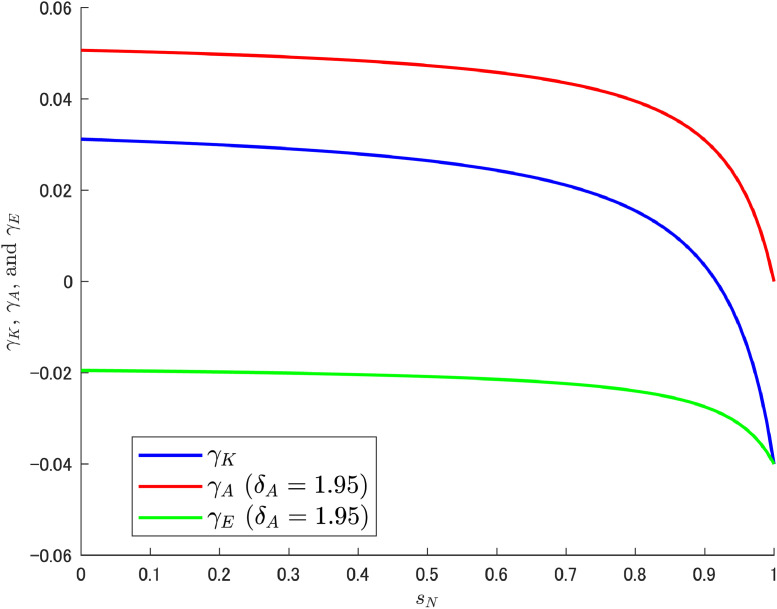
Relationship among $\gamma_{K}$, $\gamma_{A}$, and $\gamma_{E}$ under $\delta_{A}=1.95$. Note: Author’s calculation.

**Fig. 4:**
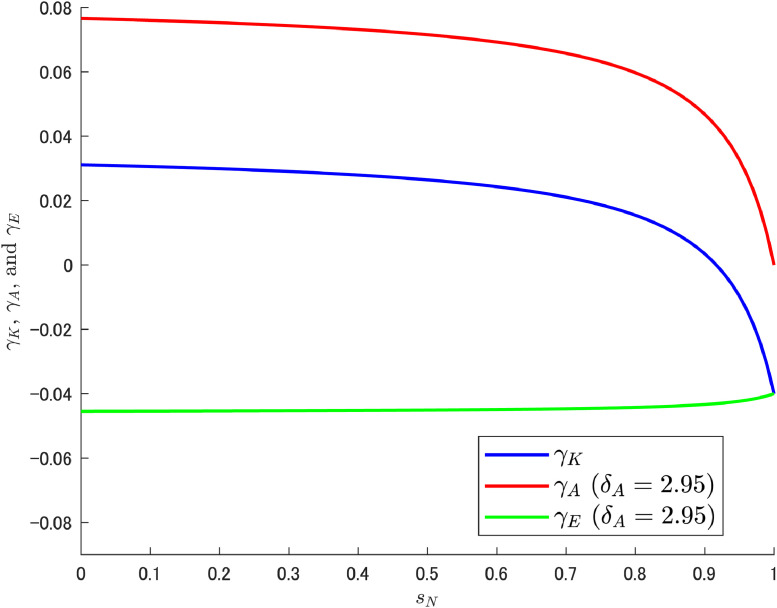
Relationship among $\gamma_{K}$, $\gamma_{A}$, and $\gamma_{E}$ under $\delta_{A}=2.95$. Note: Author’s calculation.

**Fig. 5:**
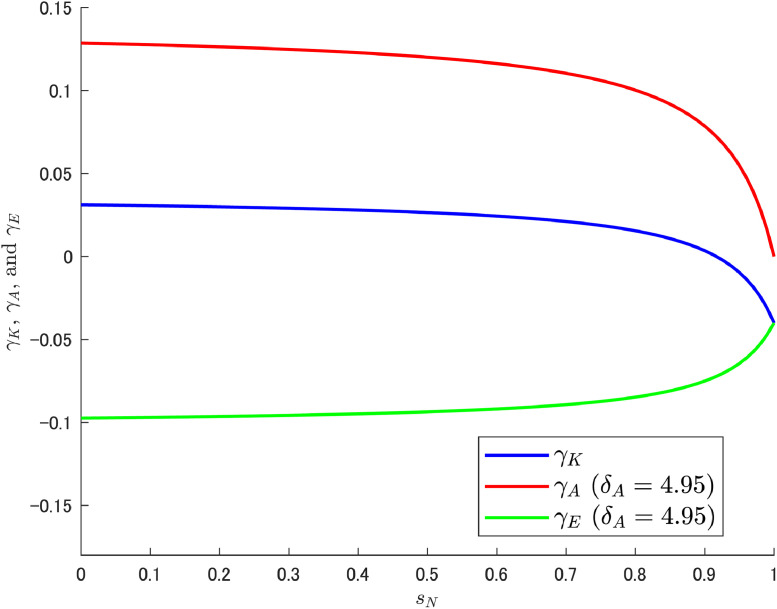
Relationship among $\gamma_{K}$, $\gamma_{A}$, and $\gamma_{E}$ under $\delta_{A}=4.95$. Note: Author’s calculation.

Let us use these equations to decompose $\gamma_{E}$ and see how they relate to $s_{N}$ under particular values of $\delta_{A}$. As noted earlier, Cheng et al. [1] considered the three cases of $\delta_{A}=\left\{0.95,\;1.95,\;2.95\right\}$, but here we add the even larger case of $\delta_{A}=4.95$, and Figs. 2; 3; 4; 5 show the results for each of these four cases.

Fig. 2, Fig. 3, Fig. 4, Fig. 5 yield some important findings. Although the growth rate of abatement technological knowledge ($\gamma_{A}$) is decreasing (i.e., downward slope) for an appropriate range of $s_{N}$, the decreasing rate of $\gamma_{A}$ is larger when $\delta_{A}$ is high and the variety-expanding R&D subsidy rate becomes particularly high. This reinforces the increasing trend of $\gamma_{E}$ (see Fig. 4, Fig. 5).^8^ Interestingly, $\delta_{A}$ itself has no relationship with labor reallocation induced by changes in $s_{N}$ (i.e., $\delta_{A}$ does not affect the amount of abatement labor), and the results are derived directly from the structural characteristics of the abatement R&D sector.

Next, we consider the impact of changing the share of abatement expenditures in GDP (β). If this share is large, then an increase in the subsidy rate for variety-expanding R&D activity would result in a substantial decrease in abatement labor. According to Cheng et al. [1], when β=0.03, the dynamic pattern of $\gamma_{E}$ changes drastically and the rate of reduction in pollution emissions slows for an increase in $s_{N}$. Following the above discussion on the impact of $\delta_{A}$, we should clarify the influence of increased subsidy to variety-expanding R&D on labor reallocation, and in particular on abatement labor. As can be seen from (4), β affects $L_{A}$, which is the major difference from the case of $\delta_{A}$.

**Fig. 6:**
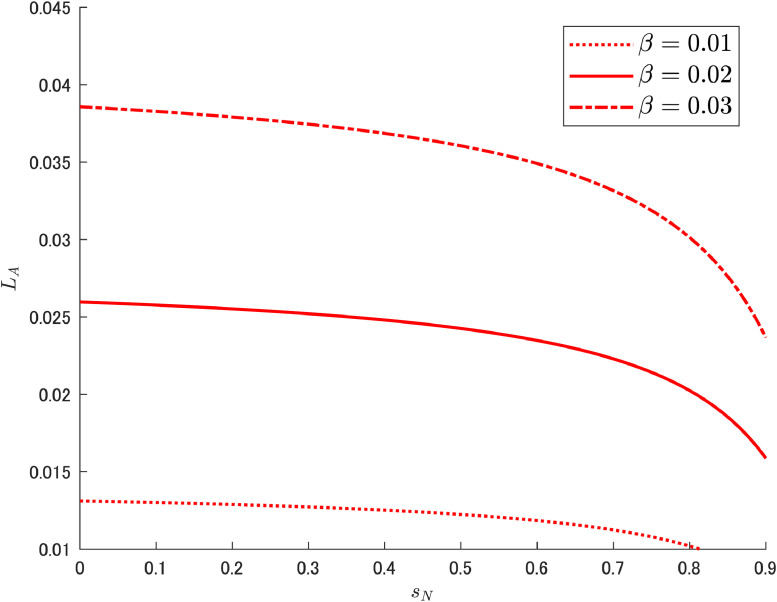
Relationship between R&D subsidy and abatement labor under different values of β. Note: Author’s calculation.

We now vary β={0.01,0.02,0.03} to see the impact, and Fig. 6 shows two important features. First, in each case there is a decreasing trend (downward slope) for an appropriate range of $s_{N}$, and this is due to the labor reallocation effect. Second, with respect to the share of abatement expenditure, the higher the government sets the share, the more abatement labor is engaged in abatement R&D; this result is due to the improvement of overall working conditions in the abatement R&D sector by enhancing the expenditure share. Taken together, the amount of abatement labor declines as the competing variety-expanding R&D sector becomes more attractive via an increase in the subsidy rate $s_{N}$, while β affects such a change in labor allocation.

**Fig. 7:**
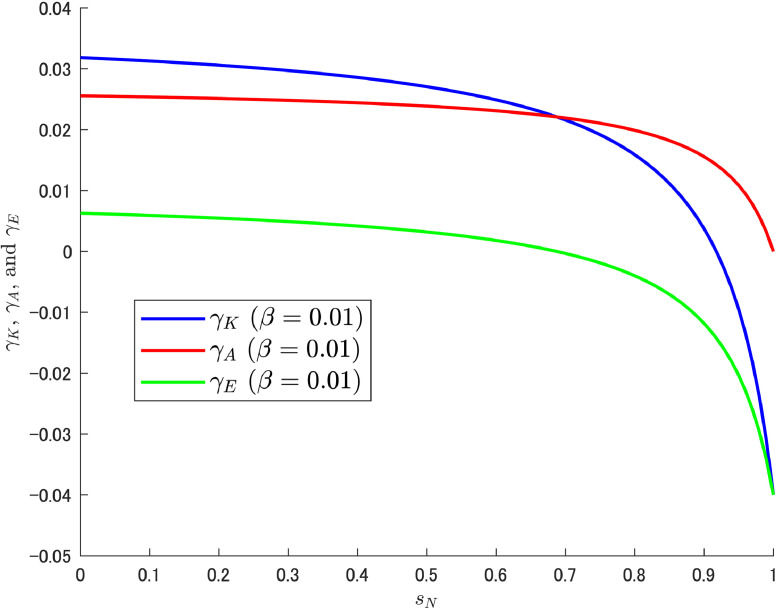
Relationship among $\gamma_{K}$, $\gamma_{A}$, and $\gamma_{E}$ under β=0.01. Note: Author’s calculation.

**Fig. 8:**
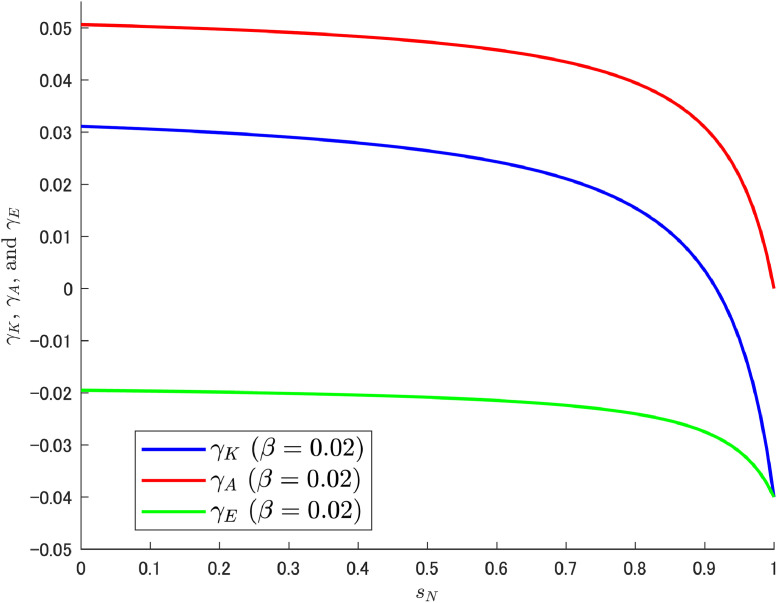
Relationship among $\gamma_{K}$, $\gamma_{A}$, and $\gamma_{E}$ under β=0.02. Note: Author’s calculation.

**Fig. 9:**
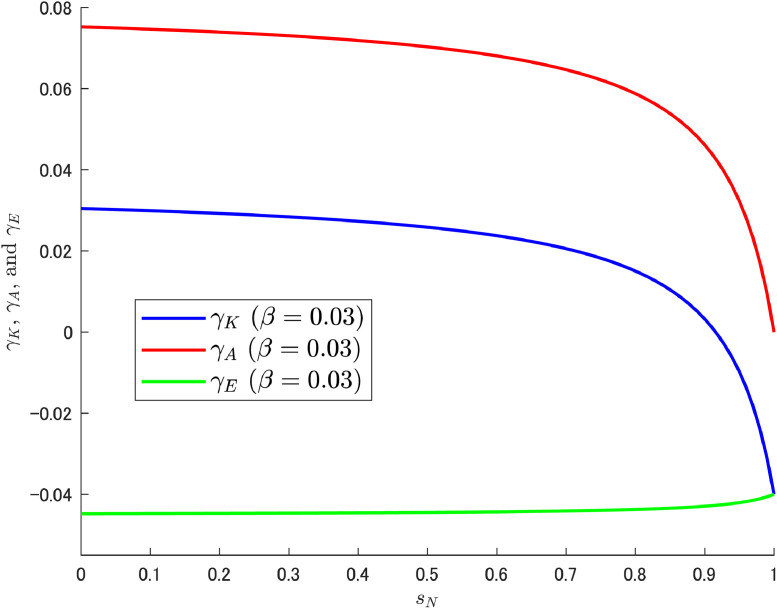
Relationship among $\gamma_{K}$, $\gamma_{A}$, and $\gamma_{E}$ under β=0.03. Note: Author’s calculation.

Let us attempt a decomposition of the growth rates (i.e., $\gamma_{K}$, $\gamma_{A}$, and $\gamma_{E}$) in the same way as in the previous $\delta_{A}$ case. As shown in Cheng et al. [1], we can expect the increasing trend of $\gamma_{E}$ to emerge at high β (0.03), which may be caused by the motion of $L_{A}$ shown in Fig. 6. The results of the decomposition under different values of β are shown in Fig. 7, Fig. 8, Fig. 9.

**Fig. 10:**
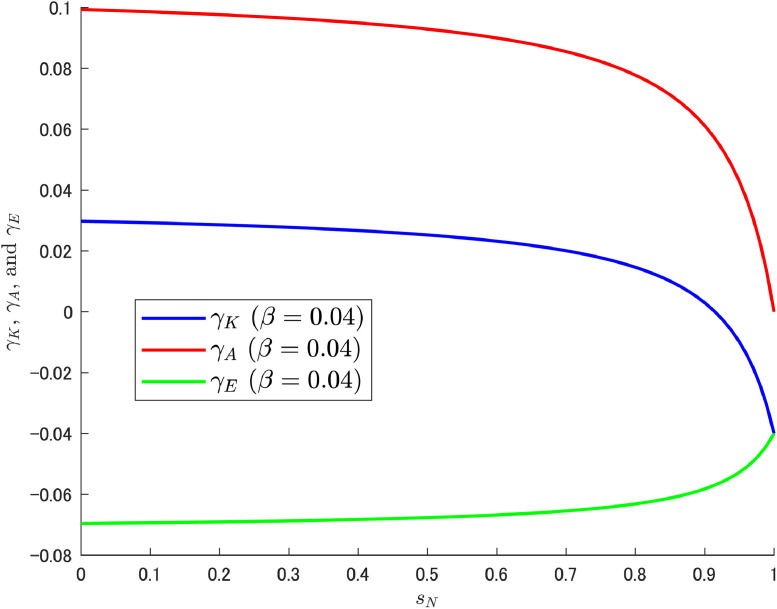
Relationship among $\gamma_{K}$, $\gamma_{A}$, and $\gamma_{E}$ under β=0.04. Note: Author’s calculation.

Fig. 7, Fig. 8 show that the change in the growth rate of abatement knowledge ($\gamma_{A}$) is moderate over most of the ranges of $s_{N}$; this is related to the relatively small value of β. Consequently, the pollution growth rate is negative and the environmental quality improves. In contrast, in Fig. 9, a relatively large value of β amplifies the effect of the change in $s_{N}$, which results in a larger change in $\gamma_{A}$. As a result, a gradual but increasing trend in $\gamma_{E}$ emerges as in Cheng et al. [1]. The increasing trend should become clearer when the share of abatement expenditure is increased further, and a case with β=0.04 (twice the calibrated value with reference to the U.S.) is shown additionally in Fig. 10.

In overview, the obtained results show that as $s_{N}$ is raised, the appeal of abatement R&D work fades and thus $L_{A}$ decreases gradually, and the effect is more pronounced for larger β (an amplification effect due to the nature of the multiplication).^9^ This has a direct effect on $\gamma_{A}$, with a large share of expenditure on abatement R&D leading to a large reduction in the growth rate of abatement knowledge in the high range of $s_{N}$. Reflecting such a situation, $\gamma_{E}\;\left(=\gamma_{K}-\gamma_{A}\right)$ then increases.

Accordingly, depending on the situation faced, strengthening the subsidy for variety-expanding R&D as a growth policy may incur high environmental costs. Consequently, a double dividend cannot be enjoyed in this case.

### On the turning point of $\gamma_{E}$

In this subsection, we examine in detail the situation in the boundary region where the sign of $\frac{\partial \gamma_{E}}{\partial s_{N}}$ changes. Specifically, we make a few comments from a policy perspective while using numerical analysis to clarify the turning point at which $\gamma_{E}$ changes from a decreasing function to an increasing function.

**Fig. 11:**
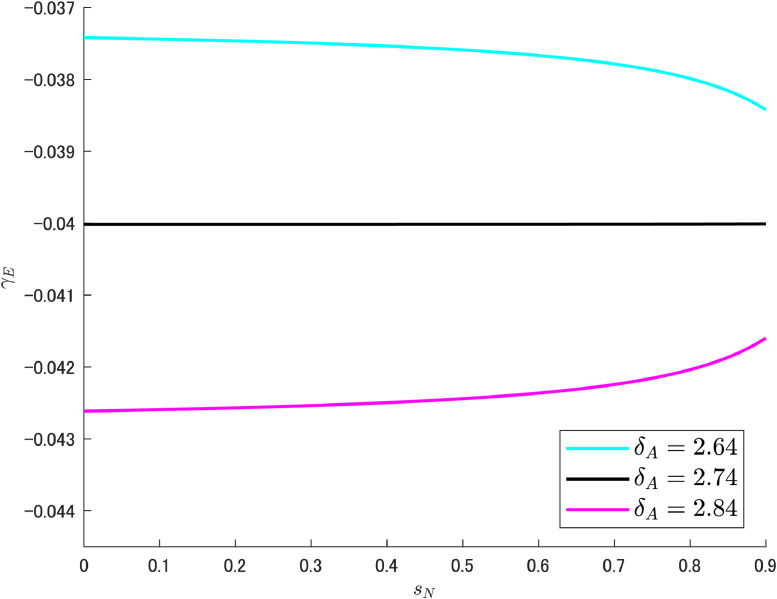
Relationship between R&D subsidy and pollution growth rates under different values of $\delta_{A}$. Note: Author’s calculation.

Fig. 11 shows the case for $\delta_{A}$. For the given parameter set, $\delta_{A}=2.74$ can be confirmed to be the turning boundary value for $\gamma_{E}$. This value is considerably larger than the benchmark value of 1.95, so $\gamma_{E}$ would exhibit a decreasing nature in the standard situation. This means that typically a double dividend would be available. Nonetheless, even in the case of an increasing trend, the change is in the negative value range, and the lack of a double dividend in the strictest sense does not seriously harm the overall performance of the economy and the environment. Although there remains little room for any intervening policies, the worries of policy makers in the current situation are likely to be minor.

**Fig. 12:**
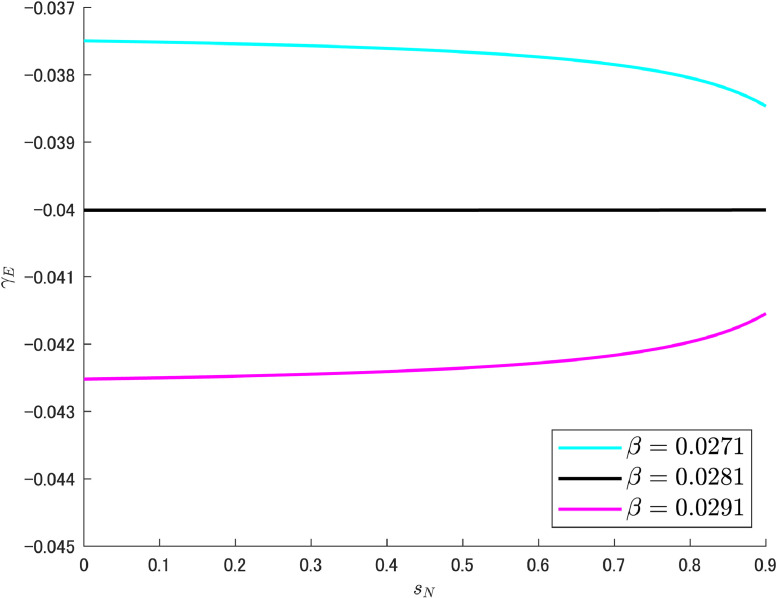
Relationship between R&D subsidy and pollution growth rates under different values of β. Note: Author’s calculation.

Next, Fig. 12 clarifies the case of β. The boundary value is 2.81%, which differs from the benchmark value (2%) by less than 1%. Because such a small difference in the targeted parameter changes the situation drastically, this seems to be a *sensitive* case that requires careful handling by the government if the reduction of pollution emissions is to be given the utmost importance. For example, if the government sets the share of abatement R&D expenditure at 3%, $\gamma_{E}$ becomes similar to the pink curve in Fig. 12, making it impossible to enjoy a double dividend in a strictest sense. However, as shown in Fig. 11, any change in $\gamma_{E}$ occurs within the negative value region, and therefore an inherently unfavorable situation is unlikely to emerge.

### Other critical issues in Cheng et al. [1]

We have discussed particularly important issues in Cheng et al. [1], but there are several other interesting issues, even if limited to those that are closely related to this paper. We would like to briefly mention a few of them here.

**Fig. 13:**
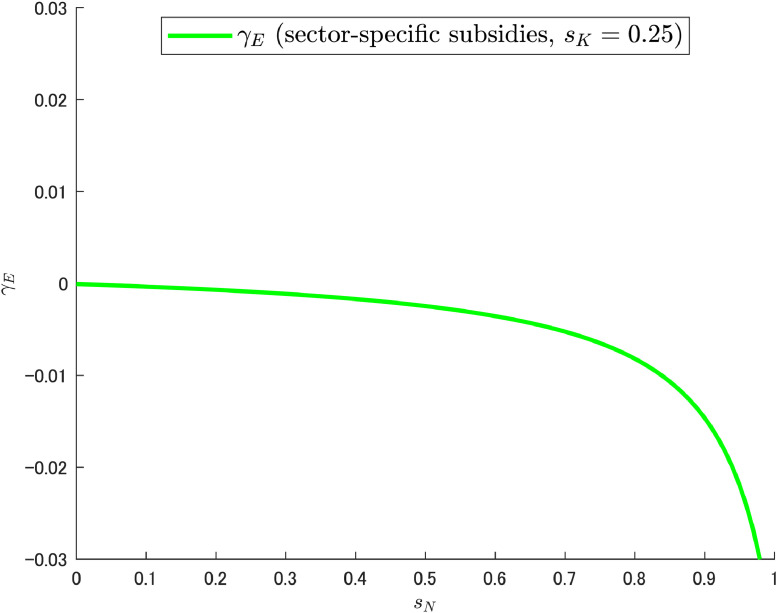
Relationship between R&D subsidy and pollution growth rates under $s_{K}\neq 0$. Note: Author’s calculation.

When considering the impact of subsidies for variety R&D on economic growth and pollution-emission rates, subsidies to capital also have an effect. Similarly, when considering how subsidies to capital affect economic and environmental performance, subsidies for variety R&D have an influence as well. Thus, the two subsidy policies are complementary to each other in obtaining a double dividend with respect to the economy and the environment. In this regard, Cheng et al. [1] performed a numerical analysis following Judd [13] and Chamley [14], in which $s_{K}=0$ was adopted as the benchmark value.^10^ Although subsidy (or taxation) on capital is an important issue and deserves to be examined in detail in a future paper, it is worthwhile to examine the situation under a relaxed assumption within the scope of Cheng et al. [1]. From the perspective of achieving a double dividend, the issue at hand is still $\gamma_{E}$. Let us now look at an interesting example. Fig. 13 shows the relationship between $s_{N}$ and $\gamma_{E}$ when $s_{K}=0.25$. It should be noted that even if $s_{K}$ is changed to negative or positive values, the downward sloping shape remains unchanged. This can be considered a favorable result for reducing pollution emissions and achieving the double dividend. However, if $s_{K}$ is increased further, the curve shifts upward toward the positive $\gamma_{E}$ region. This means that, given the current combination of parameters, there is an upper bound on the value of $s_{K}$ for the growth rate of pollution emissions to be negative and the double dividend to be achieved in the strictest sense.

Next, we would like to comment on the results shown in Fig. 6. Compared with changes in the share of public expenditure on abatement R&D, abatement labor has shifted to a relatively large extent. This shift in labor is closely related to the mechanism discussed in this paper and is theoretically an intriguing point. However, when considering the characteristics of people working in the abatement R&D sector, it remains to be examined whether such a shift can occur smoothly in the real world or not. For example, one possibility would be to incorporate barriers to labor mobility across sectors, taking into account differences in human capital levels.

The households in Cheng et al. [1] have a utility function that derives utility from consumption but experiences disutility from pollution flows, so it is important to perform a welfare analysis, which they did not attempt. However, a detailed analysis of welfare is beyond the scope of this paper and will be left to a future paper, but some interesting inferences can be made from similar studies that implement two growth engines, such as Chang et al. [15]. Chang et al. [15] performed a detailed welfare analysis, showing that as pollution taxes increase, welfare levels increase monotonically. Of particular interest is that when the parameter representing the extent of households’ environmental concerns in the utility function is relatively small, nonlinearity emerges between pollution taxes and welfare. However, when concerns about the environment are high, this parameter value becomes larger, the nonlinearity mentioned above disappears, and a monotonically increasing relationship is obtained. Because the utility functions of Chang et al. [15] and Cheng et al. [1] are essentially the same, it is expected that similar interesting results will be obtained when welfare analysis is attempted in line with the model of Cheng et al. [1].^11^

Finally, we would like to note the dynamic properties of the original model. In general, dynamic macroeconomic models that include environmental factors often exhibit interesting equilibrium dynamics (see e.g., [16]). What happens in an environmental macroeconomic model that implements two growth engines? In the case of Cheng et al. [1], the long-term steady-state is dynamically unstable, and the economy has no transitional dynamics. This is a characteristic feature often seen in AK models ([17]), and similar equilibrium dynamics have been reported in the environmental macroeconomics literature, including Chu et al. [18].

## Conclusion

We have complemented the analysis of Cheng et al. [1] by presenting a detailed mechanism for the cases in which the double dividend fails to hold in a strict sense in terms of economic growth and pollution emissions (i.e., environmental quality). In particular, we found that the share of public spending on environmental abatement R&D activities can easily change the resulting situation compared with the benchmark value (the hardship of the double dividend). Practically, it is important to keep this result in mind when promoting environmental R&D societally.

## Ethics statements

Not applicable.

## Supplementary material *and/or* additional information [optional]

None.

## CRediT authorship contribution statement

**Kei Hosoya:** Conceptualization, Formal analysis, Funding acquisition, Methodology, Software, Validation, Visualization, Writing – original draft, Writing – review & editing.

## Declaration of competing interest

The author declares no known competing financial interests or personal relationships that could have appeared to influence the work reported in this paper.

## Footnotes

1 Capital goods are inputs for producing intermediate goods, and the variety of such intermediate goods increases via variety-expanding R&D. Thus, both the accumulation of capital and the increase in the variety of intermediate goods are engines of growth. Similar to Cheng et al. [1], others that implemented multiple growth engines include Chu et al. [19], Beladi et al. [20], and Chang et al. [15].

2 In line with the model proposed by Chu and Lai (2014), Hosoya [21] performed a numerical analysis to clarify the importance of efficiency in the R&D sector as a complementary factor to environmental taxes.

3 For the economic growth rate $\gamma_{Y}$, both subsidy and patent policies generally result in $\gamma_{Y}>0$ in a given situation, so that one of the double dividends holds.

4 The notation here is described as necessary; see Cheng et al. [1] for further details.

5 Although the policy of strengthening patent protection also yields a double dividend, our focus is on the more dynamically interesting case of subsidy policy.

6 From a mathematical perspective, β seemingly affects the shape of the curve itself.

7 See Appendix A.3 (Proof of Lemma 1) in Cheng et al. [1].

8 The increase in $\gamma_{E}$ occurs within the negative value region, meaning that the rate of reduction in pollution emissions slows down.

9 Note that the amplification effect arising in $L_{A}$ was not found in $\delta_{A}$.

10 For details on this issue, see also Lucas [22].

11 In Cheng et al. [1], the utility function is a log-linear type, and the extent of households’ concern for the environment (i.e., pollution emission flows) is captured by η.
